# Validity Evidence for the New American Board of Emergency Medicine Certifying Examination

**DOI:** 10.1002/aet2.70159

**Published:** 2026-03-30

**Authors:** Michael Gottlieb, Madison Holzman, Kevin B. Joldersma, Kathleen C. Ruff, Susan E. Farrell, Felix K. Ankel, John L. Kendall, Melissa A. Barton, Diane L. Gorgas

**Affiliations:** ^1^ American Board of Emergency Medicine East Lansing Michigan USA; ^2^ Department of Emergency Medicine Rush University Medical Center Chicago Illinois USA; ^3^ Holzman Consulting Staunton Virginia USA; ^4^ Department of Emergency Medicine Brigham and Women's Hospital Boston Massachusetts USA; ^5^ Department of Emergency Medicine Regions Hospital St. Paul Minnesota USA; ^6^ Department of Emergency Medicine, Stanford School of Medicine Stanford University Standford California USA; ^7^ Department of Emergency Medicine Ohio State College of Medicine Columbus Ohio USA

## Abstract

**Introduction:**

Certification of emergency physicians includes a two‐phase process, including both a written qualifying examination and an oral certification examination. In 2026, the American Board of Emergency Medicine will institute a new certifying examination (CE), which will be an in‐person assessment with objective structured clinical examinations (OSCEs), including focused communication‐centered cases and procedural assessments. The objective of this paper is to provide initial validity evidence for the new CE.

**Methods:**

A unified view of validity was used to guide the collection of evidence for content validity for the new CE. Moreover, an evaluation of possible validity threats was conducted. An iterative and collaborative process including focus groups, a summit, one‐on‐one employer interviews, and public and patient input was used to identify key domains and establish the exam blueprint for the new CE. The exploratory phase for exam development, known as the Becoming Certified Initiative, spanned a two‐year timeline. Evidence was collected at a practice administration of the CE through the evaluation of candidates' and examiners' perceptions of alignment of case content to emergency medicine (EM) practice, as well as understanding and clarity of the cases and their instructions.

**Results:**

The new CE blueprint comprises 10 components: four clinical cases covering clinical decision‐making and prioritization and six OSCEs covering procedures, ultrasound, patient‐centered communication, reassessment, difficult conversations (delivering unwanted or unexpected information), and managing conflict. Both candidate and examiner volunteers rated the cases as clinically accurate and relevant to EM practice. They also reported that case instructions and prompts within the cases were generally clear and understandable.

**Conclusion:**

This study provides important early validity evidence for the new CE to inform emergency physicians and the public at large.

## 1 Introduction

Certification of emergency medicine (EM) physicians by the American Board of Emergency Medicine (ABEM) includes a two‐phase process. Candidates must first pass the written Qualifying Examination (QE), which assesses their knowledge of The Model of the Clinical Practice of Emergency Medicine (EM Model) core content. The QE is followed by the Oral Certification Examination (OCE), which evaluates clinical decision‐making and skills unique to the EM environment.

Attaining ABEM certification confers a measure of trust that the diplomate can demonstrate the knowledge and skills required of a practicing emergency physician. As such, it is critical for physicians, hospital administrators, and the public to have confidence in the certification process, including transparency regarding key changes and their rationale, as well as the provision of its supporting validity evidence.

Prior to recognition by the American Board of Medical Specialties (ABMS) in 1979, ABEM published early validity evidence for its OCE [1]. The initial ABEM oral examination was innovative in that it introduced criterion referencing (vs. normative referencing) into the scoring process. Criterion referencing uses a passing score established through a psychometric process called standard‐setting; thus, any test taker performing at or above this predetermined threshold will pass the examination. In 2015, ABEM introduced the enhanced oral (eOral) examination [2]. The eOral format used a computer monitor that enhanced the visual fidelity of the assessment experience with elements such as audio and video stimuli and continuously running vital signs that change throughout the case. In 2020, the Structured Interview case type was added to enhance the OCE's ability to reveal clinical decision‐making.

While the existing OCE emphasized cognitive skills, ABEM recognized the need for a more comprehensive assessment tool to identify additional competencies, including communication, procedural, ultrasound, and prioritization skills. To address these needs, ABEM will institute a new certifying examination (CE) in 2026. The new CE will include a substantially different examination approach through in‐person assessment with objective structured clinical examinations (OSCEs) and procedural assessments. The objective of this paper is to provide initial validity evidence for this new CE.

## 2 Methods

### 2.1 Competency Framework Selection

Several ABMS Member Boards, including the American Board of Surgery and the American Board of Pediatrics, have adopted Entrustable Professional Activities (EPAs) as the primary outcome competency for certification assessment. In contrast, ABEM elected not to adopt a standalone EPA‐based framework for the CE as EPAs have not permeated into EM training to the degree in which they have been implemented for other specialties, such as surgery and pediatrics. Moreover, ABEM believes a different framework is necessary to support the nature of EM practice and the episodic, high‐stakes nature of decisions that must often be made quickly across concurrent tasks. ABEM continues to work collaboratively to explore how EM EPAs within residency might better align with board eligibility outcome competencies.

Instead of EPAs, ABEM grounded the CE blueprint in the 2022 EM Model and the ABMS Standards for Initial Certification, using a domain‐and‐component structure that allows direct assessment of observable performance across high‐stakes encounters. While several components of the CE align conceptually with EPA constructs (e.g., independent management of acutely ill patients, communication during critical conversations), ABEM determined that a domain‐based framework better supported a defensible, standardized assessment for use in an OSCE‐based certifying examination.

### 2.2 Study Design

**FIGURE 1:**
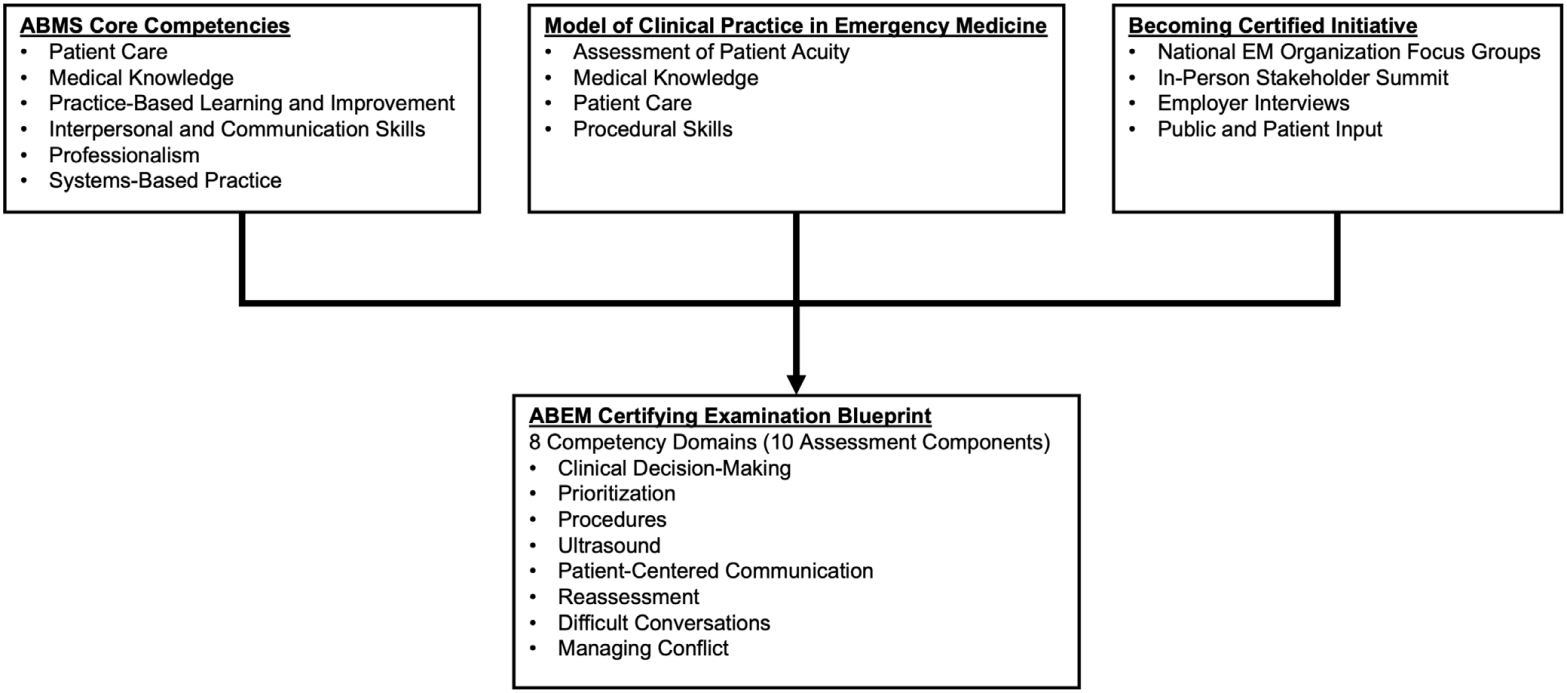
Conceptual framework guiding development of the American Board of Emergency Medicine certifying examination blueprint. ABEM, American Board of Emergency Medicine; ABMS, American Board of Medical Specialties; EM, Emergency Medicine.

This study focused on gathering initial validity evidence for the new ABEM CE. It is commonly accepted in medical testing to consider validity as a unitary concept that uses the accumulation of various types of validity evidence to support score interpretation and use, rather than claiming a test either has or does not have validity [3, 4]. As such, to guide collection of validity evidence for the new CE, validity was considered a unitary concept, where accumulation of evidence is used to support the claim that candidates passing the CE can demonstrate the core competencies and knowledge to practice EM. As the CE is a new exam and has not been formally opened for candidate testing, this study focused primarily on collecting foundational validity evidence related to content, as well as preliminary evidence to rule out threats to validity. This study focuses on CE blueprint development guided by the Becoming Certified Initiative (BCI) [5] and early evidence of mitigation of construct irrelevant variance for candidates and examiners, specifically regarding clarity and understanding of case content, case administration instructions, and the scoring instructions. Our validity argument presented herein reflects a multistep process, proceeding from blueprint development through the collection of early validity evidence during testing (Figure 1). This study was deemed exempt from human research by the West‐Copernicus Group Institutional Review Board.

The development of a testing blueprint is foundational to content validity, as it defines the construct and the subsequent test used to measure it [6]. The ABEM CE blueprint development process was iterative and collaborative. First, the 2022 EM Model was evaluated to identify potential domains for the new CE to focus on [7]. This was further informed by the ABMS Standards for Initial Certification [8]. The initial domains were determined by reviewing the items in both elements line by line and mapping them against the existing OCE. After identifying initial domains, ABEM began the work for the BCI by leading focus groups in early 2022 with EM organization leaders [9]. This helped identify the core competencies of a certified emergency physician. Findings from focus groups involving a wide array of stakeholders, in conjunction with the EM Model, informed the planning of an in‐person summit with EM leaders and interest‐holders in March 2022. The purpose of this in‐person summit was to determine how ABEM should assess physicians given the changing landscape of healthcare and EM as a specialty. Finally, in August 2022, a third‐party consulting firm conducted interviews of emergency physician employers to ensure their representation was incorporated into the identification of test content. Employer interviews were used to elicit the competencies or factors considered when hiring emergency physicians, as well as factors most often cited for termination. The CE blueprint was developed using findings from each stage of the BCI as described and was approved by ABEM's Board of Directors. After cases were developed based on the blueprint, a practice administration was held in June 2025 to pilot the CE and to evaluate the cases and blueprint.

### 2.3 Study Setting and Population

The BCI team invited leaders from core EM organizations to participate in virtual focus groups. Thirty‐nine leaders representing seven organizations participated: Association of Academic Chairs of Emergency Medicine, American Academy of Emergency Medicine, American Academy of Emergency Medicine Resident and Student Association, American College of Emergency Physicians, Council of Residency Directors in Emergency Medicine, Emergency Medicine Residents' Association, and Society for Academic Emergency Medicine. Each organization provided between three and nine representatives.

During the next phase of the BCI, 55 participants (34 physicians, 18 non‐physician organizational leaders, and 3 patient representatives) representing areas relevant to EM convened for an in‐person summit. Participating groups included community and academic emergency physicians, EM residents, EM residency program directors, EM department chairs, hospital leaders, patient representatives, public advisors, EM organizational leaders, ABMS staff, and ABEM staff. Patient representatives were selected based upon prior experience working with ABEM and ABMS. Characteristics of the physicians attending the BCI summit are included in Appendix A. Employer interviews were conducted virtually by a third‐party consulting firm, and participants included 29 individuals who employ or work alongside emergency physicians. During the two‐year BCI, over 4300 stakeholders provided feedback on content and structure for the exam.

The BCI informed the blueprint that was used to develop the CE. A practice administration of the CE was conducted on‐site at the Assessment, Innovation, Measurement, and Excellence Center in Raleigh, North Carolina where the CE will be administered. The administration took place over 2 days. The setting and experience were structured to closely resemble the eventual CE experience. ABEM invited individuals who currently serve as oral examiners to participate in the practice administration as either candidates or examiners. Both examiners and candidates were chosen to represent a diversity of practice, time since initial certification, and active participation status in the CE case development process. All are current ABEM volunteers, but none of the candidates had previous involvement with the development process or knowledge of the content of the new CE. In total, 30 currently board‐certified individuals participated as either candidates or examiners.

### 2.4 Study Protocol

During the BCI phase, focus groups were convened for one‐hour, semi‐structured sessions that were conducted virtually. Each focus group had between five and 20 participants, and individuals from different organizations were represented within each group. For the list of questions, see Appendix B. After each of the focus groups, ABEM staff identified common themes and used these themes to inform the next stages of blueprint development. The staff conducted a thematic synthesis of the qualitative findings, grouping by common elements. ABEM staff worked with ABEM directors to arrive at the initial topic groups. Non‐physician ABEM staff were intentionally selected to enhance reflexivity in theme selection. Themes were considered salient when they emerged independently across multiple stakeholder groups and aligned with expectations for unsupervised EM practice rather than based on frequency counts alone.

The in‐person summit was unstructured and used to build upon or further understand themes identified from the focus groups. This summit was also used to gather additional insights which had not yet been identified in the blueprint development process. All employer interviews were conducted one‐on‐one with an employer and consultant from the third‐party consulting firm. The interviews were semi‐structured, virtual, and lasted approximately 1 hour.

After the construction of the CE, ABEM held a CE practice administration. All candidates and examiners participating in the practice administration completed an electronic survey via Qualtrics (Qualtrics LLC; Seattle, WA), rating various aspects of the cases. Specifically, participants were asked to evaluate the relevance and accuracy of the cases to EM. Evaluating cases for relevance and accuracy to a domain is a common practice to gather content validity evidence [5]. Candidates were asked questions such as “The cases were clinically accurate to emergency medicine practice” and “The case topics were relevant to the practice of emergency medicine.” Examiners were asked “Were the cases clinically accurate of emergency medicine practice?” and “How would you rate the relevance of the case topic to the specialty?” For all cases, candidates and examiners were provided with the case topic and used it when responding to survey questions. That is, when evaluating the cases, candidates and examiners understood how the questions aligned to the domains outlined in the CE blueprint.

To identify potential sources of construct irrelevant variance, candidates and examiners were asked questions related to timing and clarity of CE cases. When collecting validity evidence, it is common and necessary to explore whether exam features or content are interfering with the intended engagement with the exam construct [3]. In this study we specifically evaluated case clarity, timing, and scoring instruction clarity. Candidates were asked questions such as “Did you understand what you were asked to do based on the task sheet provided in hallway prior to entering the room?”; “Did you have sufficient time to review the task sheet in the hallway for each case prior to entering the room?”; “The examiners' prompts, when applicable, were clear and understandable”; and, where relevant, “The standardized patient actors' prompts were clear and understandable.” Examiners were asked questions including “I understood how to administer the case” and “How clear were the scoring instructions?”. For the clinical decision‐making and prioritization cases, the examiner provided the instructions directly, so no task sheet questions were asked. While these questions do not ensure candidates or examiners engaged with the CE constructs as intended, they do help to surface instances where the intended CE score interpretations are at risk due to timing or clarity (i.e., construct irrelevant features of the examination). For the list of survey items, see Appendices C and D.

Following the practice administration, separate focus groups of examiners and candidates were conducted. Examiners and candidates were asked to provide qualitative feedback about the case administration and areas of improvement needed, the degree to which the case assessed the particular competency, and whether the examiner and candidate believed that the case format aligned with the performance expectations for an ABEM‐certified physician. These findings were analyzed by ABEM staff and physicians using a similar thematic analysis as described above. Surveys and focus groups were intentionally performed among candidates in addition to examiners to obtain an independent perspective and triangulate findings of what occurred during the administration rather than relying solely on examiner perception.

### 2.5 Data Analysis

For the focus group, in‐person summit, and employer interviews held during the BCI, consistent themes were identified, and these findings were triangulated across phases to develop the CE blueprint. Practice administration survey data were analyzed descriptively within the Qualtrics survey software, and data were reported as counts and frequencies. As the sample size is relatively small, percentages can be easily misinterpreted and are not reported.

## 3 Results

Key themes identified from the focus groups, in‐person summit, and employer interviews of the BCI are provided in Table 1. Clinical skills and procedural competencies were identified across all three groups. Communication also consistently arose as a competency necessary for certification and hiring, as well as a reason emergency physicians are often terminated. Findings also pointed to the idea that communication is a multifaceted and complex topic that includes patients, an emergency physician's team, and difficult conversations.

**TABLE 1:** Key themes elicited during blueprint development via the BCI.

Focus group themes (competencies for an ABEM‐certified physician)	In‐person summit (assessing physicians)	Employer interviews (competencies for hiring and reasons for termination)
Procedural competency (kinesthetics) Procedural indications, risks, and benefits Trouble‐shooting procedural complications Professionalism Judgment Making difficult decisions Addressing undifferentiated clinical presentations Problem solving in a complex situation Integrating new and evolving information Using consultants from other specialties Multi‐tasking/Code‐switching Team communication Social aspects (social determinants of health) Cultural competency Patient advocacy	Complex communications Assessing patient‐centric communication and relationship skills Assessing procedural competencies Use of simulation	*Hiring factors* ABEM certification Medical knowledge Clinical skills Clinical experience Communication skills *Termination factors* Inability to lead a team Poor interpersonal communication

Abbreviations: ABEM, American Board of Emergency Medicine; BCI, Becoming Certified Initiative.

Based on triangulated findings from the BCI, the EM Model, and expert consensus, the ABEM Board of Directors approved a CE blueprint consisting of eight competency domains operationalized through 10 assessment components. For purposes of the CE blueprint, domains represent broad competency areas derived from the EM Model and stakeholder input, while components represent discrete, observable assessment units within each domain. Domains are assessed through single examination stations (e.g., clinical cases, OSCEs). Components constitute the operational elements of the CE blueprint and each component corresponds directly to a single domain. Table 2 displays the mapping of each component to its corresponding domain, illustrating how the blueprint translates abstract competency areas into observable performance assessments. These 10 components included four clinical cases covering clinical decision‐making and prioritization, and six OSCEs covering procedures, ultrasound, patient‐centered communication, reassessment, difficult conversations, and managing conflict. The number of components for each domain was determined by the importance of the skill as identified during the blueprint development process.

**TABLE 2:** Certifying examination blueprint.

Clinical cases	Number	Objective structures clinical exams	Number
Clinical decision‐making	2	Procedure	1
Prioritization	2	Ultrasound	1
		Patient‐centered communication	1
		Reassessment	1
		Difficult conversation	1
		Managing conflict	1
Session total cases*	4	Session total cases*	6
Testing time total (minutes)	60	Testing time total (minutes)	60

* Clinical cases are 15 min each; OSCEs are 10 min each.

At the practice administration, most candidates and examiners reported the cases had strong alignment regarding clinical accuracy and relevancy to the practice of EM (Table 3). When asked about the clarity of cases, the majority of candidates indicated they understood what they were asked to do for most cases and had sufficient time to review the task sheet and materials prior to beginning the case (Table 4).

**TABLE 3:** Candidate and examiner ratings of case clinical accuracy and relevancy.

Candidate evaluations					
Question	Domain	Completely	Mostly	Somewhat	No
The cases were clinically accurate to emergency medicine	Clinical decision‐making (*N* = 27)	18	6	1	2
	Prioritization (*N* = 24)	17	7	0	0
	Procedure (*N* = 20)	17	2	1	0
	Ultrasound (*N* = 20)	15	4	1	0
	Reassessment (*N* = 23)	20	3	0	0
	Difficult conversations (*N* = 21)	18	3	0	0
	Managing conflict (*N* = 21)	17	4	0	0
	Patient‐centered Communication (*N* = 20)	19	1	0	0
The case topics were relevant to the practice of emergency medicine	Clinical decision‐making (*N* = 27)	22	3	0	2
	Prioritization (*N* = 24)	22	2	0	0
	Procedure (*N* = 20)	17	2	1	0
	Ultrasound (*N* = 20)	15	3	2	0
	Reassessment (*N* = 23)	20	2	1	0
	Difficult conversations (*N* = 21)	20	1	0	0
	Managing conflict (*N* = 21)	19	2	0	0
	Patient‐centered communication (*N* = 20)	19	1	0	0

Examiner evaluations					
Question	Domain	Completely	Mostly	Somewhat	Not at all
Were the cases clinically accurate of emergency medicine practice?	Clinical Decision‐making (*N* = 10)	8	2	0	0
	Prioritization (*N* = 12)	11	1	0	0
	Procedures (*N* = 2)	1	1	0	0
	Ultrasound (*N* = 2)	2	0	0	0

Question	Domain	Very relevant	Relevant	Somewhat relevant	Not relevant
How would you rate the relevance of the case topic to the specialty?	Clinical Decision‐making (*N* = 10)	8	2	0	0
	Prioritization (*N* = 12)	12	0	0	0
	Procedure (*N* = 2)	2	0	0	0
	Ultrasound (*N* = 2)	2	0	0	0

**TABLE 4:** Candidate evaluations of case clarity and time to review materials.

Domain	Question	Yes	Somewhat	No
Procedure (*N* = 20)	Did you understand what you were asked to do based on the task sheet provided in the hallway prior to entering the room?	19	1	0
	Did you have sufficient time to review the task sheet in the hallway for each case prior to entering the room?	20	0	0
Ultrasound (*N* = 20)	Did you understand what you were asked to do based on the task sheet provided in the hallway prior to entering the room?	15	4	1
	Did you have sufficient time to review the task sheet in the hallway for each case prior to entering the room?	19	1	0
Reassessment (*N* = 23)	Did you understand what you were asked to do based on the task sheet provided in the hallway prior to entering the room?	19	3	1
	Did you have sufficient time to review the task sheet in the hallway for each case prior to entering the room?	21	2	0
Difficult Conversations (*N* = 21)	Did you understand what you were asked to do based on the task sheet provided in the hallway prior to entering the room?	18	3	0
	Did you have sufficient time to review the task sheet in the hallway for each case prior to entering the room?	21	0	0
Managing Conflict (*N* = 21)	Did you understand what you were asked to do based on the task sheet provided in the hallway prior to entering the room?	19	2	0
	Did you have sufficient time to review the task sheet in the hallway for each case prior to entering the room?	21	0	0
Patient‐centered Communication (*N* = 20)	Did you understand what you were asked to do based on the task sheet provided in the hallway prior to entering the room?	18	2	0
	Did you have sufficient time to review the task sheet in the hallway for each case prior to entering the room?	20	0	0

*Note:* For the purposes of the administration, the task sheet refers to the intake form provided prior to entering the case room.

When asked about their experiences with examiners' prompts and/or standardized patient‐actors' prompts, most candidates indicated that the prompts they received were clear and understandable for nearly all cases (Table 5a). The highest number reporting disagreement was for ultrasound (*n* = 5). When asked to rate the clarity and their experience with administration instructions and scoring instructions, examiners generally indicated agreement regarding clarity and comfort with the materials (Table 5b). Nearly all examiners indicated they understood how to administer the case. Examiners did, however, indicate some uncertainty with scoring the clinical decision‐making and prioritization cases.

**TABLE 5a:** Candidate experiences with examiners' and/or standardized patient actors' prompts.

Domain	Question	Strongly agree	Agree	Disagree	Strongly disagree	N/A
Clinical Decision‐making (*N* = 27)	The examiners' prompts, when applicable, were clear and understandable	10	13	1	1	2
Prioritization (*N* = 24)	The examiners' prompts, when applicable, were clear and understandable	6	16	2	0	0
Procedure (*N* = 20)	The examiners' prompts, when applicable, were clear and understandable	10	9	1	0	0
Ultrasound (*N* = 20)	The examiners' prompts, when applicable, were clear and understandable	9	6	5	0	0
Reassessment (*N* = 23)	The standardized patient actors' prompts were clear and understandable	11	12	0	0	0
Difficult Conversations (*N* = 21)	The standardized patient actors' prompts were clear and understandable	13	8	0	0	0
Managing Conflict (*N* = 21)	The examiners' prompts, when applicable, were clear and understandable	10	8	0	0	3
	The standardized patient actors' prompts were clear and understandable	14	7	0	0	0
Patient‐centered Communication (*N* = 20)	The standardized patient actors' prompts were clear and understandable	12	8	0	0	0

Abbreviation: N/A, not applicable.

**TABLE 5b:** Examiner experiences with case and scoring instructions.

Domain	Question	Strongly agree	Agree	Disagree	Strongly disagree
Clinical Decision‐making (*N* = 10)	I understood how to administer the case.	8	1	0	1
Prioritization (*N* = 12)		9	3	0	0
Procedure (*N* = 2)		2	0	0	0
Ultrasound (*N* = 2)		2	0	0	0

Domain	Question	Completely	Mostly	Somewhat	Not at all
Clinical Decision‐making (*N* = 10)	How clear were the scoring instructions?	0	6	3	1
Prioritization (*N* = 12)		0	7	2	3
Procedure (*N* = 2)		0	1	1	0
Ultrasound (*N* = 2)		0	2	0	0

*Note:* Responses limited to those who performed assessments in person. Examiner experience data for asynchronous scorers was not available.

## 4 Discussion

This study represents the first step in gathering validity evidence for the new ABEM CE. Using a structured, iterative process that incorporated perspectives from national EM organizations, academic and community leaders, employers, and patients/public stakeholders, we developed a CE blueprint and piloted a CE that reflects the competencies expected of a modern board‐certified emergency physician. Our findings demonstrate strong alignment of case content with the EM Model [6], with both candidate and examiner volunteers rating the cases as clinically accurate and relevant to EM practice. Moreover, participants reported that case instructions and examiner or standardized patient prompts were generally clear and understandable, thereby providing evidence that the validity of the CE exam scores is not limited due to case clarity or timing.

Our results emphasize the importance of engaging diverse stakeholders in the development of high‐stakes certification examinations. Themes identified during the BCI across focus groups, the summit, and employer interviews converged on competencies that extend beyond traditional medical knowledge, with particular emphasis on procedural ability and communication. Notably, communication was a recurrent theme across all groups and reinforced prior literature highlighting poor communication as a leading cause of adverse outcomes, disciplinary action, and physician termination [10].

The development of the ABEM CE aligns with broader trends across ABMS Member Boards toward performance‐based assessment while reflecting important specialty‐specific distinctions. Similar to other specialties such as the American Board of Anesthesiology and the American Board of Urology [11, 12], ABEM has shifted away from exclusive reliance on knowledge‐based testing toward direct observation of clinical performance through structured, standardized encounters. Unlike Boards that have adopted EPAs as the primary organizing framework, ABEM elected to employ a domain‐and‐component model anchored in the EM Model. This approach parallels the use of OSCE‐based, domain‐driven assessments in other acute care specialties and supports the episodic, high‐stakes nature of EM practice, where entrustment decisions must often be made rapidly and across multiple concurrent tasks. Importantly, while not explicitly framed as EPAs, many CE components function analogously by requiring candidates to integrate medical knowledge, procedural skill, communication, and professional judgment within a single assessment encounter. The ABEM approach thus represents an alternative but conceptually aligned pathway to competency‐based certification that prioritizes feasibility, standardization, and defensibility in a national certifying examination.

The CE practice administration provided preliminary support for content validity and that case instructions and design do not detract from candidates' engagement with the CE construct domains as intended. Moreover, while this study did not specifically evaluate whether examiners scored the cases equitably or as intended, findings do suggest that at this time examiners' engagement with the scoring instructions is not limited by their clarity for most cases. Candidates and examiners largely agreed that the cases were clinically accurate and relevant, and most reported clear understanding of the case instructions and prompts. However, areas for refinement remain. Candidates suggested that instructions for some cases, particularly prioritization and ultrasound, could be clarified further. Similarly, examiner feedback highlighted uncertainty regarding the scoring of prioritization cases. These findings provide valuable insight to enhance and further calibrate future iterations of the CE. For example, there is now one case each for difficult conversations, managing conflict, and patient‐centered communication.

The evolution of ABEM's assessment strategy mirrors broader trends in physician certification, which increasingly emphasize demonstration of clinical judgment, procedural competence, and communication skills in addition to knowledge acquisition [11]. Early validity work on the ABEM OCE established the importance of criterion‐referenced standard‐setting to ensure fairness and defensibility of pass/fail decisions [1]. More recently, the transition to the eOral examination highlighted the role of fidelity and authenticity in assessment [2]. The new CE extends this trajectory by incorporating OSCEs and direct assessment of procedural, ultrasound, and communication skills. The emphasis on domains such as difficult conversations, patient‐centered communication, and shared decision‐making reflects the realities of modern EM, where competence extends beyond knowledge and technical skill to include judgment, adaptability, and interpersonal effectiveness.

## 5 Limitations

This paper has several limitations. First, this study only provided evidence for content validity and explored how case timing and clarity may influence the CE. Although examining how timing and clarity pose sources of construct irrelevant variance may be discussed under the umbrella of response process evidence [3], we did not collect direct evidence to evaluate the extent to which candidates or examiners engaged with the constructs and case materials as intended. Direct examination of candidate reasoning relative to intended constructs will require cognitive interviewing or think‐aloud methods in future studies. After CE examination administration officially begins, future work will focus on collecting other validity evidence such as relations to key outcomes and related variables, internal evidence of the CE, and consequences of testing. Second, the practice administration involved a small number of participants, which may limit the generalizability of the findings. Third, examiner data for the practice administration were only available for those who performed the assessments in person. A subset of assessments was performed via asynchronous scoring; however, this group did not receive post‐examination surveys. Finally, this study was limited to a single practice administration of the examination with volunteer candidates, and future work should collect this evidence across a larger number of administrations.

## 6 Conclusion

To further align the certification of emergency physicians to today's medical needs, ABEM will institute a new CE. Through the BCI, a collaborative process including a wide array of key stakeholders, ABEM identified important areas on which new physicians must demonstrate proficiency to be successful in their specialty. These areas are represented on the CE blueprint, which ABEM used to develop clinical cases and OSCEs for the in‐person CE. Content validity evidence is supported through the development of the CE blueprint, and preliminary evidence to rule out validity threats related to case clarity and timing is supported through the evaluation of candidates' and examiners' understanding of case content and instructions. As validity is an accumulation of evidence, this work represents a first step in the collection of validity evidence for the new CE.

## Author Contributions

Study Concept and design: M.G., M.H., K.C.R., M.A.B., D.L.G. Acquisition of the data: M.H., K.B.J. Analysis and interpretation of the data: M.G., M.H. Drafting the manuscript: M.G., M.H., K.B.J., K.C.R., S.E.F., F.K.A., J.L.K., M.A.B., D.L.G. Critical revision of the manuscript: M.G., M.H., K.B.J., K.C.R., S.E.F., F.K.A., J.L.K., M.A.B., D.L.G. Statistical expertise: M.G., M.H.

## Funding

The authors have nothing to report.

## Conflicts of Interest

Dr. Gottlieb, Dr. Joldersma, Ms. Ruff, and Dr. Barton are employees of the American Board of Emergency Medicine (ABEM). Drs. Farrell, Ankel, Kendall, and Gorgas serve on the ABEM Board of Directors. ABEM receives revenue from the Certifying Examination.

## Supporting information


**Data S1:** aet270159‐sup‐0001‐Supinfo.docx.

## Data Availability

The data that support the findings of this study are available on request from the corresponding author. The data are not publicly available due to privacy or ethical restrictions.
