# Supplementary material for: Validity Evidence for the New American Board of Emergency Medicine Certifying Examination
Source: AEM Educ Train. 2026 Mar 30;10(2):e70159. doi: 10.1002/aet2.70159 (PMC13557208; doi:10.1002/aet2.70159)
Supplement: Supplementary file 1 [file AET2-10-e70159-s001.docx]

**Supplemental Materials.**

**Appendix D. Physician Characteristics of Becoming Certified Initiative Panel**

| **Demographics** | **Physician Characteristics (n=34)** |
| --- | --- |
| Years in Practice (mean [SD]) | 24 years (SD: 13 years) |
| Gender (n, %)  Man  Woman  Not Reported | 22 (64.7%)  9 (26.5%)  3 (8.8%) |
| Region  Midwest  Northeast  South  West  Not Reported | 11 (32.4%)  9 (26.5%)  3 (8.8%)  10 (9.4%)  1 (2.9%) |
| Practice Location  Metro/Urban  Not Metro/Urban  Not Reported | 24 (70.6%)  5 (14.7%)  5 (14.7%) |
| Practice Type  Academic Medical Center  Community Hospital  Not Reported | 17 (50.0%)  12 (35.3%)  5 (14.7%) |

**Appendix B. Focus Group Interview Guide**

1. What is the value of board certification to the Emergency Medicine specialty and community?
2. What do you think ABEM should say to the public and patients about ABEM certified physicians?
3. What are important messages for emergency physicians to hear about initial certification?
4. What should individuals who hire emergency physicians know about the value of ABEM certified physicians?
5. What should an emergency physician be expected to do as a certified physician? What should be the goals and priorities for emergency physician competence that would influence the design of the initial certification process?
6. Residency graduates take a written, qualifying exam, and a virtual oral exam to become certified. What is important to be maintained in initial certification?
7. What does the current certification process not assess sufficiently?
8. Given new educational and learning modalities, what methods and/or models would you suggest ABEM explore in redesigning initial certification?
9. What are potential opportunities to collaborate regarding initial certification?

**Appendix C. Practice Administration Candidate Surveys^1^**

Clinical Decision Making

1. Did you understand what you were asked to do based on the task sheet provided in the hallway prior to entering the room?
2. Did you have sufficient time to review the task sheet in the hallway for each case prior to entering the room?
3. The examiners’ prompts, when applicable, were clear and understandable.
4. The cases were clinically accurate to emergency medicine practice.
5. The case topics were relevant to the practice of emergency medicine.

Prioritization

1. Did you understand what you were asked to do based on the task sheet provided in the hallway prior to entering the room?
2. Did you have sufficient time to review the task sheet in the hallway for each case prior to entering the room?
3. The examiners’ prompts, when applicable, were clear and understandable.
4. The cases were clinically accurate to emergency medicine practice.
5. The case topics were relevant to the practice of emergency medicine.

Reassessment

1. Did you understand what you were asked to do based on the task sheet provided in the hallway prior to entering the room?
2. Did you have sufficient time to review the task sheet in the hallway for each case prior to entering the room?
3. The standardized patient actors’ prompts were clear and understandable.
4. The cases were clinically accurate to emergency medicine practice.
5. The case topics were relevant to the practice of emergency medicine.

Difficult Conversations

1. Did you understand what you were asked to do based on the task sheet provided in the hallway prior to entering the room?
2. Did you have sufficient time to review the task sheet in the hallway for each case prior to entering the room?
3. The standardized patient actors’ prompts, when applicable, were clear and understandable.
4. The cases were clinically accurate to emergency medicine practice.
5. The case topics were relevant to the practice of emergency medicine.

Managing Conflict

1. Did you understand what you were asked to do based on the task sheet provided in the hallway prior to entering the room?
2. Did you have sufficient time to review the task sheet in the hallway for each case prior to entering the room?
3. The examiners’ prompts, when applicable, were clear and understandable.
4. The standardized patient actors’ prompts, when applicable, were clear and understandable.
5. The cases were clinically accurate to emergency medicine practice.
6. The case topics were relevant to the practice of emergency medicine.

Patient-centered Communication

1. Did you understand what you were asked to do based on the task sheet provided in the hallway prior to entering the room?
2. Did you have sufficient time to review the task sheet in the hallway for each case prior to entering the room?
3. The standardized patient actors’ prompts, when applicable, were clear and understandable.
4. The cases were clinically accurate to emergency medicine practice.
5. The case topics were relevant to the practice of emergency medicine.

Procedures

1. Did you understand what you were asked to do based on the task sheet provided in the hallway prior to entering the room?
2. Did you have sufficient time to review the task sheet in the hallway for each case prior to entering the room?
3. The examiners’ prompts, when applicable, were clear and understandable.
4. The cases were clinically accurate to emergency medicine practice.
5. The case topics were relevant to the practice of emergency medicine.

Ultrasound

1. Did you understand what you were asked to do based on the task sheet provided in the hallway prior to entering the room?
2. Did you have sufficient time to review the task sheet in the hallway for each case prior to entering the room?
3. The examiners’ prompts, when applicable, were clear and understandable.
4. The cases were clinically accurate to emergency medicine practice.
5. The case topics were relevant to the practice of emergency medicine.

*^1^, Presented survey items represent those relevant to the collection of content and response process validity evidence. Additional questions were asked to evaluate the cases and administration processes more robustly.*

**Appendix D. Practice Administration Examiner Survey for Clinical Decision Making, Prioritization, Ultrasound, & Procedures^1^**

1. I understood how to administer the case.
2. Were the cases clinically accurate of emergency medicine practice?
3. How would you rate the relevance of the case topic to the specialty?
4. How clear were the scoring instructions?

*^1^, Presented survey items represent those relevant to the collection of content and response process validity evidence. Additional questions were asked to evaluate the cases and administration processes more robustly.*
